# Vitamin D status of Arab Gulf residents screened for SARS-CoV-2 and its association with COVID-19 infection: a multi-centre case–control study

**DOI:** 10.1186/s12967-021-02838-x

**Published:** 2021-04-26

**Authors:** Nasser M. Al-Daghri, Osama E. Amer, Naif H. Alotaibi, Dara A. Aldisi, Mushira A. Enani, Eman Sheshah, Naji J. Aljohani, Naemah Alshingetti, Suliman Y. Alomar, Hanan Alfawaz, Syed D. Hussain, Abdullah M. Alnaami, Shaun Sabico

**Affiliations:** 1 Chair for Biomarkers of Chronic Diseases, Biochemistry Department, College of Science, King Saud University, PO Box, 2455, Riyadh, 11451 Saudi Arabia; 2 Department of Medicine, College of Medicine, King Saud University, Riyadh, 12372 Saudi Arabia; 3 Department of Community Health Sciences, College of Applied Medical Sciences, King Saud University, Riyadh, 11451 Saudi Arabia; 4 Infectious Diseases Section, King Fahad Medical City, Riyadh, 59046 Saudi Arabia; 5 Diabetes Care Center, King Salman Bin Abdulaziz Hospital, Riyadh, 12769 Saudi Arabia; 6 Obesity, Endocrine and Metabolism Center, Department of Medicine, King Fahad Medical City, Riyadh, Kingdom of Saudi Arabia; 7 Obstetrics and Gynaecology Department, King Salman Bin Abdulaziz Hospital, Riyadh, 11564 Saudi Arabia; 8 Doping Research Chair, Department of Zoology, College of Science, King Saud University, Riyadh, 11495 Saudi Arabia; 9 College of Food Science & Agriculture, Department of Food Science & Nutrition, King Saud University, Riyadh, 11495 Saudi Arabia

**Keywords:** Covid-19, Vitamin D, Case–control, Saudi

## Abstract

**Objectives:**

Vitamin D status in patients with COVID-19 is an on-going controversial issue. This study aims to determine differences in the serum 25(OH)D concentrations of Arab Gulf adult residents screened for SARS-CoV-2 and its association with risk of COVID-19 infection together with other comorbidities.

**Methods:**

In this multi-center, case–control study, a total of 220 male and female adults presenting with none to mild symptoms were screened for COVID-19 (n = 138 RT-PCR-confirmed SARS-CoV-2 positive and 82 negative controls). Medical history was noted. Anthropometrics were measured and non-fasting blood samples were collected for the assessment of glucose, lipids, inflammatory markers and serum 25(OH)D concentrations.

**Results:**

Serum 25(OH)D levels were significantly lower in the SARS-CoV-2 positive group compared to the negative group after adjustment for age and BMI (52.8 nmol/l ± 11.0 versus 64.5 nmol/l ± 11.1; p = 0.009). Being elderly (> 60 years) [Odds ratio 6 (95% Confidence Interval, CI 2–18; p = 0.001) as well as having type 2 diabetes (T2D) [OR 6 (95% CI 3–14); p < 0.001)] and low HDL cholesterol (HDL-c) [OR 6 (95% CI 3–14); p < 0.001)] were significant risk factors for COVID-19 infection independent of age, sex and obesity.

**Conclusions:**

Among Arab Gulf residents screened for SARS-CoV-2, serum 25(OH) D levels were observed to be lower in those who tested positive than negative individuals, but it was the presence of old age, diabetes mellitus and low-HDL-c that were significantly associated with risk of COVID-19 infection. Large population-based randomized controlled trials should be conducted to assess the protective effects of vitamin D supplementation against COVID-19.

**Supplementary Information:**

The online version contains supplementary material available at 10.1186/s12967-021-02838-x.

## Introduction

Infection from the Severe Acute Respiratory Syndrome Coronavirus-2 (SARS-CoV-2), the causative pathogen of the coronavirus disease-2019 (COVID-19), has clinical consequences ranging from mild flu-like illness to severe pneumonia with acute respiratory distress syndrome (ARDS) which can further progress to septic shock and death. To date, COVID-19 has infected more than a hundred million individuals from 230 nations globally, with casualties exceeding 2.8 million since its discovery [1]. The Middle East and the Gulf Cooperation Council (GCC) countries were not spared from this on-going pandemic and coincidentally, preexisting conditions linked to COVID-19 severity such as obesity [2, 3], type 2 diabetes (T2D) [4, 5], and vitamin D deficiency [6, 7] are also highly prevalent in the region, giving extra challenge to the already burdened healthcare system.

Vitamin D is a steroid hormone involved in the modulation of the innate and acquired immune system as well as in the production of the antimicrobial peptides such as human β-defensin-2 and cathelicidin, in addition to the expression of genes responsible for the destruction of the intracellular pathogens [8–10]. Many studies have consistently suggested that vitamin D deficiency is associated with increased risk of respiratory tract infections, especially in influenza and now, COVID-19 [11–14]. In fact, in the early months of the pandemic, several experts were swift to push the idea of providing vitamin D supplementation since the immune-boosting actions of vitamin D in human health are well established given its anti-viral properties [15], and that vitamin D deficiency is widespread, especially among those deemed to be at increased risk for COVID-19 morbidity and mortality such as the elderly and those with preexisting conditions [16–18]. Some of the mechanisms behind the beneficial effects of vitamin D involve inhibition of pro-inflammatory cytokines in human monocytes/macrophages [19], as well as inhibition of the Renin Angiotensin System (RAS) [20]. The angiotensin-converting enzyme 2 (ACE2) receptor, the point of entry of SARS-CoV-2 and protective against hypertension and inflammation [21, 22], is strengthened by vitamin D via heightened expression that balances ACE/ACE2 and angiotensin II (ANG)/ANG 1–7, pathways that are known to be disrupted by coronaviruses [23].

While many observational studies have demonstrated vitamin D deficiency among patients with severe COVID-19 [6, 24, 25], there is scarcity of available evidence that have attempted to differentiate differences in the vitamin D status of asymptomatic to mild cases swabbed for SARS-CoV-2 in the GCC region. To the best of our knowledge, such observational study has never been conducted within GCC and Saudi Arabia in particular. Vitamin D deficiency within the GCC region is unique in that it is highly prevalent despite all-year sunshine [26], more common in summer [27], and that internationally recommended doses at 2000 IU still produces suboptimal levels [28]. These exceptional regional features and the possible role of vitamin D in the fight against Covid-19 are interesting to investigate. A series of studies from observational to clinical trials involving participants with Covid-19 may therefore shed light whether vitamin D deficiency is a risk factor for Covid-19 infection in this population and may explain, other than the mitigation strategies employed by the government, the characteristics of the Covid-19 cases that maybe unique in the country. In the present cross-sectional study therefore, we aimed to assess differences in serum 25(OH)D levels and other clinical characteristics of Arab Gulf residents tested for COVID-19.

## Methods

### Participants

In this multi-center, case–control study, a total of 220 adult participants aged 30–60 years, residents of Riyadh, Saudi Arabia were included. These participants were swabbed for reverse transcription polymerase chain reaction (RT-PCR) confirmation of SARS-CoV-2 at King Saud University Medical City-King Khalid University Hospital (KSUMC-KKUH) and King Salman Hospital (KSH), Riyadh, Saudi Arabia, within May–July 2020 [N = 138 tested positive for SARS-CoV-2 and N = 82 tested negative as the control group]. For the purpose of this study only asymptomatic to mild cases were included, and participants with severe manifestations of COVID-19 (those that required intensive care) were excluded. According to the Ministry of Health, a mild category meant that the patient required no O_2_ on presentation, no evidence of pneumonia but with clinical symptoms such as fever [29]. Diagnosis of RT-PCR-confirmed SARS-CoV-2 was also based on national guidelines in Saudi Arabia [29].

### Anthropometry, blood collection and sample analysis

Non-fasting blood samples were collected and anthropometrics measurements include height (rounded off to the nearest 0.5 cm), weight (rounded off to the nearest 0.1 kg), blood pressure in mmHg (mean of two readings) using standard procedures. Body mass index was calculated (kg/m^2^). Fasting glucose and lipid profile including triglycerides, total cholesterol, LDL cholesterol (LDL-c), HDL-c were analyzed using a chemical analyzer (Konelab, Espoo, Finland). Total serum 25(OH)D was measured using commercial electrochemiluminescence immunoassay (Roche Diagnostics, Germany). This test has an intra and inter-assay coefficients of 4.6% and 5.3%, respectively. RT-PCR was done on nasopharyngeal samples which were obtained from patients and sent to the Biosafety Level 2-facility (BSL-2) in KSUMC, Riyadh, KSA according to manufacturer’s instructions. Vitamin D deficiency [25(OH)D < 50 nmol/l] was defined based on national and regional recommendations [28, 30].

### Sample size calculations

Sample size was calculated on the basis of a previous study undertaken in children [31] and adults [32] where the prevalence of vitamin D deficiency among cases and non-cases of severe acute lower respiratory infection was reported to be 50% and 20%, respectively. On this basis of these results, a sample size of N = 74 for each group is required (total sample size of 148) to achieve 98% significance level and 94% statistical power [31]. G*Power software was used for post-hoc power analysis [33]. Given the total sample size (N = 220) and obtained 25(OH)D levels between SARS-CoV-2 positive and negative groups yielded 100% power to detect significance.

### Data analysis

Data was entered and analyzed using SPSS version 21. Results were presented as N (%) for categorical variables and mean ± standard deviation for continuous variables. Non-normal variables were logarithmically transformed prior to all parametric analyses. Statistical differences between COVID-19 status and other categorical variables were tested using the chi-square test of independence. Bivariate associations were done to determine associations of 25(OH)D to measured parameters and comorbidities. Analysis of covariance (ANCOVA) was used to determine statistical differences after adjusting for covariates (age and BMI). Bonferroni correction (p = 0.05/n = 13) was also applied in the interpretation of differences and a p < 0.0038 was considered significant. Binary logistic regression was used to determine associations between COVID-19 status and clinical characteristics after adjusting for age, obesity and sex. Significance was set at p < 0.05.

## Results

Table 1 shows the general clinical characteristics of participants. Out of the 220 swabbed, 138 were confirmed to be SARS-CoV-2 positive (79 males and 59 females) while 82 were found to be negative (41 males and 41 females). The negative group was significantly younger and had lower BMI compared to the SARS-CoV-2 positive group (p-values < 0.001). No differences in nationalities were observed. With respect to medical history, the SARS-CoV-2 positive group had a higher prevalence of diabetes, hypertension, hyperlipidemia, hypertriglyceridemia and low-HDL than the negative group (p-values < 0.001, < 0.001, 0.04, 0.04 and < 0.001, respectively). A significantly higher prevalence of vitamin C and D supplement use was observed in the SARS-CoV-2 positive group during the pandemic (p-values < 0.001). The rest of the characteristics are found in Table 1.

**Table 1:** General Characteristics of Participants

Parameters	Overall	Negative	Positive	p-value
N (%)	220	82 (37)	138 (63)	
Anthropometrics and Demographics				
Male/Female	120/100	41/41	79/59	0.30
Age (years)	43 ± 15	32 ± 13	50 ± 13	< 0.01
BMI (kg/m^2^)	28 ± 5.5	27 ± 5	29 ± 5.5	< 0.01
Saudi	139 (63.2)	57 (70)	82 (59)	0.13
Non-Saudi	81 (36.8)	25 (30)	56 (41)	
Medical History of Patients				
Obesity	69 (33.2)	21 (28)	48 (36)	0.20
Type 2 Diabetes	102 (49.0)	14 (18)	88 (68)	< 0.01
Hypertension	53 (24.1)	5 (6)	48 (35)	< 0.01
Cancer	4 (1.8)	4 (5)	0 (0)	0.06
Rheumatoid Arthritis	5 (2.3)	2 (2)	3 (2)	0.90
Chronic Liver Disease	1 (0.5)	0 (0)	1 (1)	0.72
Thyroid Disease	5 (2.3)	2 (2)	3 (2)	0.90
Epilepsy	2 (0.9)	0	2 (1)	0.48
Chronic Immobilization	1 (0.5)	0	1 (1)	0.72
Asthma	10 (4.5)	1 (1)	9 (6)	0.10
Hyperlipidemia	14 (6.4)	0	14 (10)	0.04
Heart Disease	10 (4.5)	0	10 (7)	0.07
Anemia	3 (1.4)	3 (4)	0	0.10
Chronic Kidney Disease	5 (2.3)	0	5 (4)	0.19
Hypertriglyceridemia	66 (31.6)	18 (23)	48 (37)	0.04
Low HDL-cholesterol	134 (64.1)	28 (35)	106 (82)	< 0.01
Supplementation				
Using Vitamin C before Pandemic	1 (0.4)	1 (1)	0	0.32
Using Vitamin C during Pandemic	85 (38.6)	6 (7)	79 (57)	< 0.01
Using Vitamin D before Pandemic	3 (1.4)	3 (4)	0	0.08
Using Vitamin D during Pandemic	58 (26.4)	1 (1)	57 (41)	< 0.01

Data presented as N (%).

BMI: body mass index; HDL: high density lipoprotein

p < 0.05 considered significant

**Fig. 1:**
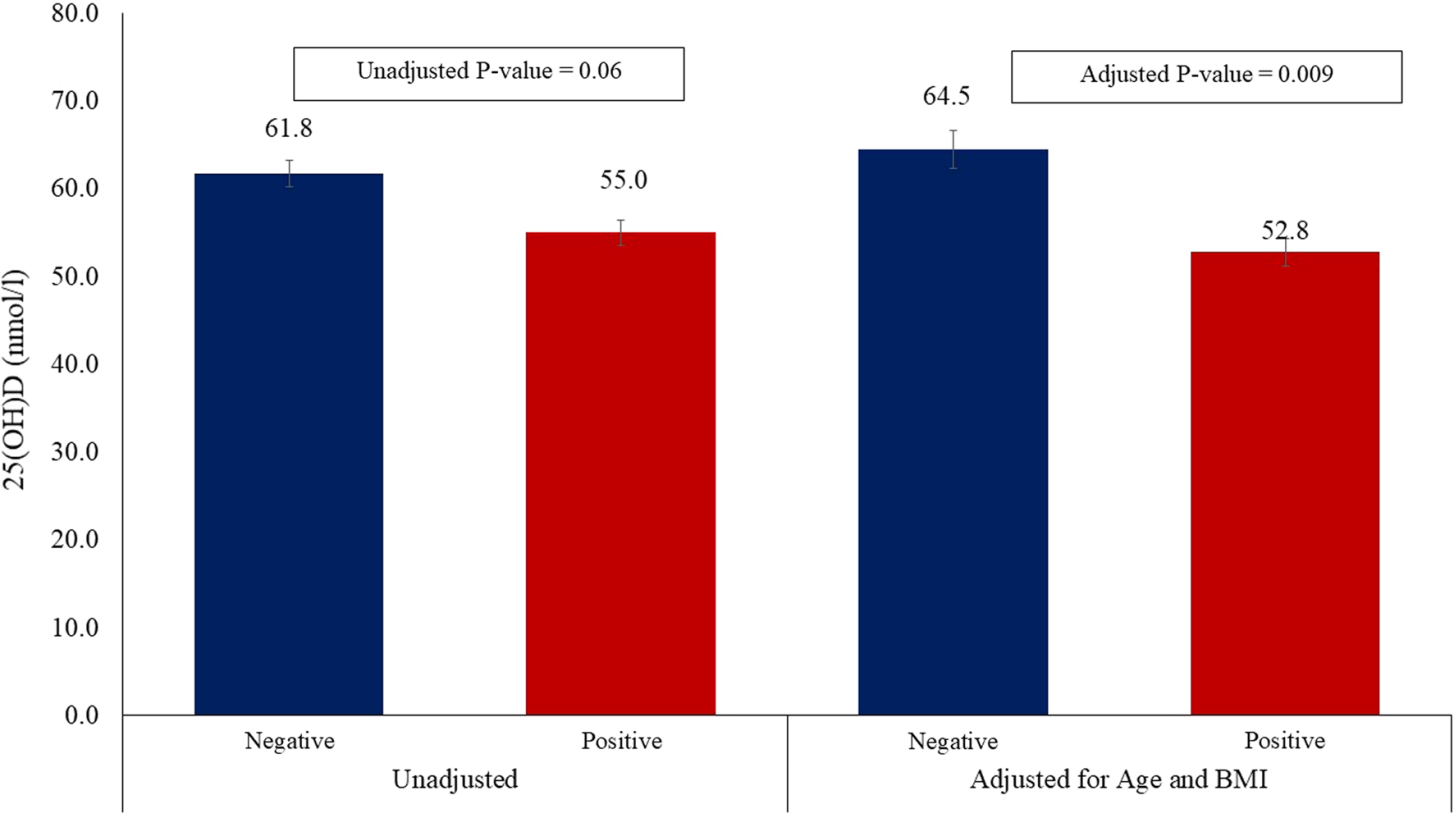
25(OH)D concentration according to SARS-CoV-2 status

Table 2 shows the unadjusted and adjusted clinical differences of both groups. The SARS-CoV-2 positive group showed a significantly worse metabolic profile than the controls. In the positive group, systolic blood pressure was significantly higher than the negative group (adjusted p < 0.001). As expected, the positive group also had a significantly higher temperature and respiratory rate on presentation (adjusted p-values < 0.001, respectively). Lipid profile was also significantly higher in the SARS-CoV-2 group than controls in terms of total cholesterol and LDL-c (adjusted p-values 0.001, respectively). HDL-cholesterol was significantly lower in the SARS-CoV-2 positive group than controls (adjusted p < 0.001). Non-fasting glucose levels was also significantly higher in the SARS-CoV-2 group as compared to controls (adjusted p = 0.004). In terms of inflammatory markers, the SARS-CoV-2 group also had significantly higher levels of CRP and IL-6 (adjusted p-values 0.008 and 0.002, respectively). Lastly, 25(OH)D levels were significantly lower in the SARS-CoV-2 group than controls after adjusting for age and BMI (adjusted p = 0.009) (Fig. 1). After applying the Bonferroni corrected p-value, only systolic blood pressure, temperature, respiratory rate, total cholesterol and CRP were significantly higher in the positive group while HDL-cholesterol was significantly lower, also in the positive group (p-values < 0.0038). The rest of the biochemical characteristics are shown in Table 2. Differences in 25(OH)D levels were analyzed using different models (Additional file 1: Table S1), showing lower but borderline significance in 25(OH)D levels in the positive group when adjusted for BMI alone (p = 0.06), but significant when adjusted for age alone (p = 0.02). Groups were further stratified according to sex, revealing no significant differences in positive and negative groups in both males (p = 0.13) and females (p = 0.32) in the unadjusted model, but borderline significance after adjusting for age and BMI (both p-values 0.06) (Additional file 1: Table S2).

**Table 2:** Clinical Characteristics of Participants According to SARS-CoV-2 Status

Clinical characteristics	Overall	R	SARS-CoV-2		p-value	p-value*
			Negative	Positive		
N	220		82 (37.3)	138 (62.7)		
Arterial Blood Pressure						
Systolic Blood Pressure (mmHg)	126 ± 16	0.02	119 ± 10	130 ± 18	< 0.001	< 0.001
Diastolic Blood Pressure (mmHg)	75 ± 11	0.00	76 ± 9	75 ± 12	0.75	0.75
Vital Signs						
Temperature (^°^C)	37.1 ± 1.1	− 0.04	36.6 ± 0.5	37.5 ± 1.2	< 0.001	< 0.001
Pulse Rate	92 ± 16	− 0.16	94 ± 15.3	92 ± 17	0.31	0.77
Respiratory Rate	22 ± 4	− 0.04	20 ± 3	24 ± 4	< 0.001	< 0.001
Lipid Profile						
Total Cholesterol (mmol/l)	4.0 ± 1.5	− 0.10	4.6 ± 1.5	3.6 ± 1.4	< 0.001	0.001
HDL-Cholesterol (mmol/l)	1.0 ± 0.4	0.02	1.2 ± 0.4	0.8 ± 0.4	< 0.001	< 0.001
LDL-Cholesterol (mmol/l)	2.4 ± 0.9	− 0.06	2.7 ± 1.1	2.0 ± 1.0	< 0.001	0.01
Triglycerides (mmol/l)	1.6 ± 1.1	− 0.07	1.4 ± 1.2	1.6 ± 1.0	< 0.001	0.22
Glycemic Profile						
Glucose (mmol/l)	9.3 ± 5.9	− 0.03	6.0 ± 3.1	10.6 ± 6.8	< 0.001	0.004
Vitamin D						
25(OH) D (nmol/L)	57.5 ± 27.5	–	61.8 ± 22.8	55.0 ± 28.8	0.06	0.009
Inflammatory Markers						
Interleukin-6 (ng/ml)	8.6 ± 11.2	0.01	6.6 ± 11.1	10.1 ± 11.1	0.004	0.008
C-Reactive Protein (µg/ml)	21.9 ± 27.7	0.02	8.9 ± 10.5	42.9 ± 33.6	< 0.001	0.002

Data presented as Mean ± SD whereas R indicates correlation coefficient, # indicates significance at 0.05. * indicates p-value adjusted for age and BMI. Bonferroni corrected p < 0.0038 is considered significant

Table 3 shows the significant risk factors for COVID-19 in the cohort, with medical comorbidities adjusted for age, sex and BMI. Age greater than 60 was 6 times more likely to test positive than those younger [OR 6.2 (95% CI 2–18); p = 0.001)]. The same risk is true for those with diabetes [adjusted OR 6.4 (95% CI 3–14); p < 0.001)] and low HDL [OR 6.1 (95% CI 3–14); p < 0.001)]. The rest of the risk factors are shown in Table 3.

**Table 3:** Unadjusted and Adjusted odds ratios for COVID-19 risk factors in the study cohort

	SARS-CoV-2		Unadjusted		Adjusted	
	Negative	Positive	OR (95% CI)	p-value	OR (95% CI)	p-value
Age > 60 years	4 (5)	33 (25)	6 (2–18)	0.001		
Male	41 (50)	79 (57)	1.3 (0.8–2)	0.3		
Obesity (BMI > 30 kg/m^2^)	21 (28)	48 (36)	1.5 (0.8–3)	0.2		
Type 2 Diabetes	14 (18)	88 (68)	10 (5–20)	< 0.001	6.4 (3–14)	< 0.001
Hypertension	5 (6)	48 (35)	8.2 (3–22)	< 0.001	2.07 (0.7–6)	0.2
Rheumatoid Arthritis	2 (2)	3 (2)	0.9 (0.1–5)	0.9	0.9 (0.1–5)	0.99
Chronic Liver Disease	0	1 (1)	1.8 (0.1–45)	0.72	–	–
Thyroid Disease	2 (2)	3 (2)	0.9 (0.1–5)	0.9	1.7 (0.2–12)	0.58
Epilepsy	0	2 (1)	3.0 (0.1–64)	0.48	–	–
Chronic Immobilization	0	1 (1)	1.8 (0.1–45)	0.72	–	–
Asthma	1 (1)	9 (6)	5.6 (0.7–45)	0.1	6.5 (0.7–61)	0.1
Hyperlipidaemia	0	14 (10)	19.2 (1.1–327)	0.04	–	–
Heart Disease	0	10 (7)	13.5 (0.8–233)	0.07	–	–
Anaemia	3 (4)	0	0.08 (0–2)	0.1	–	–
Chronic Kidney Disease	0	5 (4)	6.8 (0.4–125)	0.19	–	–
Hypertriglyceridemia	18 (23)	48 (37)	2.0 (1–4)	0.04	1.7 (0.8–4)	0.17
Low HDL-Cholesterol	28 (35)	106 (82)	8.1 (4–15)	< 0.001	6.1 (3–14)	< 0.001

Data presented as frequencies (%) and Odds Ratio (OR) (95%CI), *p* < 0.05 considered significant

## Discussion

In our study, we found that serum 25OHD levels were significantly lower in COVID-19 patients than in controls even after adjusting for the main confounding factors and that old age, diabetes and low-HDL were significantly associated with COVID-19 infection. The interaction between 25(OH)D and viral infections is a subject of increasing concern and lessons from previous similar epidemics may offer insights as to why vitamin D supplementation maybe protective against COVID-19. SARS-CoV-1 was observed to downregulate type 1 interferon (IFN) receptors which negatively affects innate immunity [34]. Unbound vitamin D receptor (VDR) weakens the protective antiviral effects of IFN via sequestration of a key transcription factor (STAT1) in IFN signaling. This inhibitory relationship between VDR and STAT1 suggests that unbinding of the latter through stimulation with biologically active forms of vitamin D (calcitriol) strengthens type 1 IFN response, which, in turn, improves innate immune system [34]. Another mechanism by which 25(OH)D can promote coronavirus degradation is through autophagy via acidification of endolysosomes, cellular organelles responsible for the release of SARS-CoV-2 in the cytosol [35, 36]. Lastly, it is now known that COVID-19 downregulates the expression of ACE2 receptors, and there is evidence that vitamin D upregulates ACE2, which can bind to SARS-CoV-2 and prevent it from binding to ACE2 receptor [37–39]. In high risk individuals, untreated COVID-19 can easily progress to cytokine storm and hyper-inflammatory state if left untreated [40]. The anti-inflammatory effects of 25(OH)D includes inhibition of tumor necrosis factor-α and IL-6 by attenuating the activation of p38 MAP kinase in human macrophages/monocytes, Additionally,1,25OH2D3 endorses the stimulation of T regulatory cells, thus inhibiting the pro-inflammatory cytokines production, including IL-21, interferon-γ, and IL-17 [41, 42].

Traditional risk factors for COVID-19 risk such as old age and T2D were also observed in the present study. The increased COVID-19 risk among those with T2D in particular was independent of age, sex and obesity status. Another risk factor however not commonly found but was identified in the present is the low- HDL-c. While low HDL-c can be partially explained by the presence of other cardiometabolic factors in the cohort such as T2D, obesity and hypertension, it’s significant association with COVID-19 highlights that aberrant lipid profiles increases susceptibility to infections [43]. HDL-c in particular has anti-inflammatory and anti-oxidative functions which, in low levels can increase pulmonary inflammation [44].

Based on the present findings, COVID-19 patients and the general population with low 25(OH) D levels serum concentrations should take vitamin D supplements, as this preventive strategy could have positive effects in boosting the immune system. Several pilot and quasi-experimental intervention studies have shown that vitamin D boluses prevent worse outcomes such as intensive care admission and better survival among the elderly [45, 46]. As more vitamin D interventional studies and clinical trials publish their results on COVID-19, it is safe to assume that doses not going beyond 2000 IU daily is beneficial for those with known vitamin D deficiency that hopefully will not be only for vitamin D status correction but more so for the prevention of acute respiratory infections, including COVID-19 [47, 48].

The authors acknowledge several limitations. First is the study design which limits interpretation as to whether low 25(OH)D status is a cause or a consequence of COVID-19 infection. Second, while the overall sample size was robust for determining differences in the clinical characteristics of participants with or without SARS-CoV2, it had low power to detect differences if stratified further according to sex which may explain the non-significant differences in males and females. The findings also apply only to individuals presenting with none to mild COVID-19 symptoms, since vitamin D status maybe an unreliable indicator in moderate to severe cases, given that it behaves as a negative acute phase reactant in the presence of a major acute inflammatory insult. Despite limitations, the present study is arguably the first to document the association of vitamin D status to COVID-19 infection among adult Arab Gulf residents screened for SARS-CoV2 and adds to the increasing call for large scale clinical trials to determine whether vitamin D correction can be used as preventive, if not therapeutic strategy against the pandemic.

## Conclusion

In summary, despite lower levels of 25(OH)D being observed among SARS-CoV2 positive cases as compared to negative controls, increased risk for COVID-19 was limited to old age, T2D and low HDL-c. The high prevalence of vitamin D deficiency in the region is enough to warrant vitamin D supplementation in the general population, but whether such strategy can be applied for the prevention of COVID-19 remains to be seen prospectively.

## Supplementary Information

**Additional file 1: Table S1**. Differences in 25(OH)D Levels Adjusted for Different Models with Associations to Age and BMI.** Table S2**. Differences in 25(OH)D Levels in Males and Females tested for SARS-Cov-2 (Unadjusted and Adjusted for Age and BMI).

## Data Availability

The datasets generated or analyzed during this study are available from the corresponding author on reasonable request.

## Abbreviations

ACE2: Angiotensin converting enzyme 2
ARDS: Acute respiratory distress syndrome
BMI: Body mass index
BSL-2: Biosafety level 2
CI: Confidence interval
COVID-19: Coronavirus disease 19
HDL-c: High density lipoprotein cholesterol
KSH: King Salman hospital
KSUMC: King Saud University Medical City
LDL-C: Low density lipoprotein cholesterol
OR: Odds ratio
RAS: Renin-angiotensin system
RT-PCR: Reverse transcriptase polymerase chain reaction
SARS-CoV-2: Severe acute respiratory syndrome coronavirus 2
