# Supplementary material for: Vitamin D status of Arab Gulf residents screened for SARS-CoV-2 and its association with COVID-19 infection: a multi-centre case–control study
Source: J Transl Med. 2021 Apr 26;19:166. doi: 10.1186/s12967-021-02838-x (PMC8072076; doi:10.1186/s12967-021-02838-x)
Supplement: Supplementary file 1 — Additional file 1: Table S1. Differences in 25(OH)D Levels Adjusted for Different Models with Associations to Age and BMI. Table S2. Differences in 25(OH)D Levels in Males and Females tested for SARS-Cov-2 (Unadjusted and Adjusted for Age and BMI). [file 12967_2021_2838_MOESM1_ESM.docx]

**Additional file 1: Table S1**. Differences in 25(OH)D Levels Adjusted for Different Models with Associations to Age and BMI

|  | **Positive** | **Negative** | **P-values** | **R** |
| --- | --- | --- | --- | --- |
| Age (years) | 50 ± 1 | 32 ± 1 | <0.001 | 0.00 |
| BMI (kg/m^2^) | 28.9 ± 0.5 | 26.6 ± 0.6 | 0.003 | 0.01 |
| 25OHD (nmol/l) | 55.0 ± 28.8 | 61.8 ± 22.8 | 0.06 |  |
| 25OHD adjusted for BMI (nmol/l) | 54.4 ± 9.4 | 61.5 ± 12.4 | 0.06 |  |
| 25OHD adjusted for age (nmol/l) | 53.6 ± 9.4 | 64.6 ± 12.4 | 0.02 |  |
| 25OHD adjusted for BMI and age (nmol/l) | 52.8 ± 11.0 | 64.5 ± 11.1 | 0.009 |  |

**Note**: Data presented as mean ± SD; R denotes correlation coefficient; significant at p<0.05.

**Additional file 1: Table S2**. Differences in 25(OH)D Levels in Males and Females tested for SARS-Cov-2 (Unadjusted and Adjusted for Age and BMI).

| **Sex** | **Positive** | **Negative** | **P-value** |
| --- | --- | --- | --- |
| **Unadjusted** | | | |
| Males | 53.1 ± 9.7 | 60.5 ± 12.3 | 0.13 |
| Females | 57.5 ± 9.7 | 62.9 ± 12.4 | 0.32 |
| **Adjusted for Age and BMI** | | | |
| Males | 51.2 ± 9.8 | 62.2 ± 12.4 | 0.06 |
| Females | 54.9 ± 9.7 | 66.7 ± 12.5 | 0.06 |

**Note**: Data presented as mean ± SD; significant at p<0.05.
